# A Review: Photonic Devices Used for Dosimetry in Medical Radiation

**DOI:** 10.3390/s19102226

**Published:** 2019-05-14

**Authors:** Edrine Damulira, Muhammad Nur Salihin Yusoff, Ahmad Fairuz Omar, Nur Hartini Mohd Taib

**Affiliations:** 1 Medical Radiation Programme, School of Health Sciences, Universiti Sains Malaysia, Kubang Kerian 16150, Malaysia; 2 Engineering Physics Laboratory, School of Physics, Universiti Sains Malaysia, Penang 11800, Malaysia; fairuz_omar@usm.my; 3 Department of Radiology, School of Medical Sciences, Universiti Sains Malaysia, Kubang Kerian 16150, Malaysia; nhartini@usm.my

**Keywords:** radiation-induced current, dosimetry, photodiodes, phototransistors (MOSFETs), photovoltaic sensor, CCD/CMOS

## Abstract

Numerous instruments such as ionization chambers, hand-held and pocket dosimeters of various types, film badges, thermoluminescent dosimeters (TLDs) and optically stimulated luminescence dosimeters (OSLDs) are used to measure and monitor radiation in medical applications. Of recent, photonic devices have also been adopted. This article evaluates recent research and advancements in the applications of photonic devices in medical radiation detection primarily focusing on four types; photodiodes – including light-emitting diodes (LEDs), phototransistors—including metal oxide semiconductor field effect transistors (MOSFETs), photovoltaic sensors/solar cells, and charge coupled devices/charge metal oxide semiconductors (CCD/CMOS) cameras. A comprehensive analysis of the operating principles and recent technologies of these devices is performed. Further, critical evaluation and comparison of their benefits and limitations as dosimeters is done based on the available studies. Common factors barring photonic devices from being used as radiation detectors are also discussed; with suggestions on possible solutions to overcome these barriers. Finally, the potentials of these devices and the challenges of realizing their applications as quintessential dosimeters are highlighted for future research and improvements.

## 1. Introduction

Radiation can be classified into mainly two, i.e., charged particle radiation that consists of fast electrons and heavy charged particles, and uncharged radiation that comprises electromagnetic radiation and neutrons [1]. In radiation spectroscopy, both these radiation types interact with matter in different ways, hence, the need for radiation measurement and monitoring in order to control its effects in the matter accordingly. Today, radiation is used for carrying out radiotherapeutic or imaging procedures like Computed Tomography (CT)—in the field of medicine [2]. However, during its applications, there has to be precaution since excessive radiation attenuation by human tissue may result into high absorbed dose values [3,4]. This could be a root cause of secondary malignancies [4]. Therefore, stringent measures to control and manage both intentional and unintentional radiation exposures through radiation dosimetry is necessary. 

Medical radiation dosimetry involves measurement, calculation, and assessment of the quantity and quality of ionizing radiation exposed to and attenuated by the human body. Various gas, liquid and solid-state dosimeters are used to quantify radiation; these are predominantly grouped under the ionization chamber, semiconductor and diamond detector types [5,6]. These detectors are used to measure radiation delivered internally (in-vivo)—by ingesting or inhaling radioactive substances, and externally using external beam radiation therapy. 

X-rays are electromagnetic waves with a wavelength shorter than that of ultra violet light but longer than that of gamma rays. X-rays, and other electromagnetic waves such as visible light, are also discrete energy packets known as photons/quanta. Quantization of X-rays could therefore make X-ray photons show characteristics of high-energy particles. In X-ray radiation detection, detection efficiency is the ratio of the number of counts in the detector’s spectrum to the number of photons emitted by the radiation source [7]. High density and the atomic number of a radiation detector material imply higher sensitivity of the detector [8]. Comparing silicon’s density; 2.3${\;\mathrm{gcm}}^{-3}$ to that of air; 1.3 × $10^{-3}{\mathrm{gcm}}^{-3}$, Romei et al. [9] state that solid-state detectors avail the same overall detection efficiency as that of gas detectors; for instance, semiconductor detectors need less energy to form an electron-hole pair compared to ionization chambers [9]. This way, applications of solid-state dosimeters in radiation detection has become recurrent in recent technologies. At an atomic level, atoms in the solid state are close to each other. On the other hand, atoms are fairly far apart from each other in gases and liquids. This makes solids to be with a higher density when compared to liquids and gases. During radiation detection, if a radiation photon strikes an atom, electrons are excited into the conduction band-hence a detectable current signal. On the other hand, when the radiation photon strikes the interatomic spacing, there will be less/no energy absorbed by the atomic electrons, hence low/no electron excitations. Consequently, there is a low detectable current signal. Therefore, a radiation photon strikes more atoms in solids than in liquids and gases. This way, solid-state detectors provide a higher resolution and sensitivity during radiation detection, i.e., if compared to liquid-state detectors and ionization chambers [2].

In particular, the semiconductor solid-state detectors mainly comprise photonic devices; circuit board components of electronic gadgets that are used for production and detection of electromagnetic radiation. Photodiodes, phototransistors, and CCDs are some of the semiconductor-based photonic devices that have profoundly been used for producing, detecting and manipulating light. Light has a wavelength range of 440–800 nm [10,11]. Nevertheless, these photonic devices are also sensitive to other ranges of the electromagnetic spectrum. Therefore, current researches aim to exploit this capability. For instance, silicon photodiodes can detect radiations having wavelengths shorter than 1.2 μm, i.e., visible and ultraviolet light, and some wavelengths near infrared radiation [12]. 

Using equations, schematics, and graphs, a detailed explication of the photonic device structure and physics is presented in the form of benefits and limitations as dosimeters. Further, factors such as minimum and maximum measurable dose, ability to give real-time measurements, reduction in sensitivity after radiation exposure, and price among others are examined. This is aimed at providing a comparative analysis of these photonic devices as medical radiation detectors since there is currently no study that has addressed this type of contradistinction.

This article, therefore, benchmarks; photodiodes, phototransistors/ MOSFETs, solar cells, and CCDs for accurate and effective medical radiation detection and measurement. This work is based on previous and current researches by different authors and ultimately aims at facilitating the development of alternative dosimeters to improvise for the conventional medical radiation dosimeters that are quite costly.

## 2. Photodiodes/LEDs

**Figure 1:**
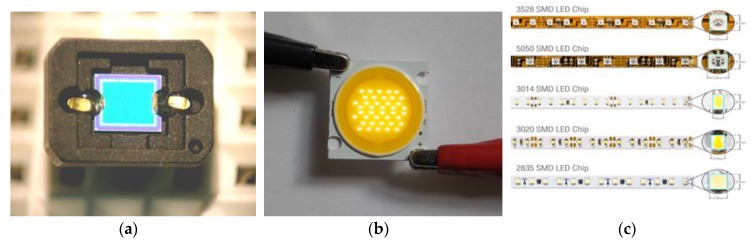
Images of some of the semiconductor-based photonic devices (**a**) Photodiode [21]; (**b**) chip on board light-emitting diode (COB LED) [22]; (**c**) surface mount diodes light-emitting diode (SMD LED) [23].

A photodiode is a semiconductor-based electrical device that converts photonic energy, in the form of electromagnetic radiation, to a detectable electrical signal in the form of current or voltage (Figure 1a). LEDs are also semiconductor-based devices. However, LEDs convert electrical energy—in the form of current, to light (photons); through the electroluminescence process [13]. To a greater extent, most of the semiconductor-based photodiodes and LEDs consist of silicon PN junctions. Today, photodiodes are typically used for signal detection [9,14,15,16] while LEDs are principally used for luminescence [13]. However, some novel studies have directly manipulated LEDs for electromagnetic radiation detection (sensing) [11,17,18,19,20]. 

Photodiodes can be broadly classified into four, i.e., PN photodiodes; consisting of a heavily positive semiconductor (P) affixed to a heavily negative semiconductor (N) to form a junction, PIN photodiodes; comprising a PN junction with an intrinsic region sandwiched between the P and N semiconductors in order to increase detection volume [14], Avalanche photodiodes (APDs): Photodiodes that are very sensitive to relatively low intensity electromagnetic radiation due to their high precision and gain capability [24], and Schottky photodiodes: Photodiodes associated with appreciably low operational capacitance [25]. 

Recent technologies deploy surface mount diodes (SMDs) and chip on board LEDs (COB LEDs). SMDs and COBs are multiple sole LEDs adhered onto a printed circuit board to form one compatible unit as shown in Figure 1b,c. COBs have more chips/LEDs, hence, provide more luminescence than SMDs; they also consume less energy [26].

### 2.1. Structure

**Figure 2:**
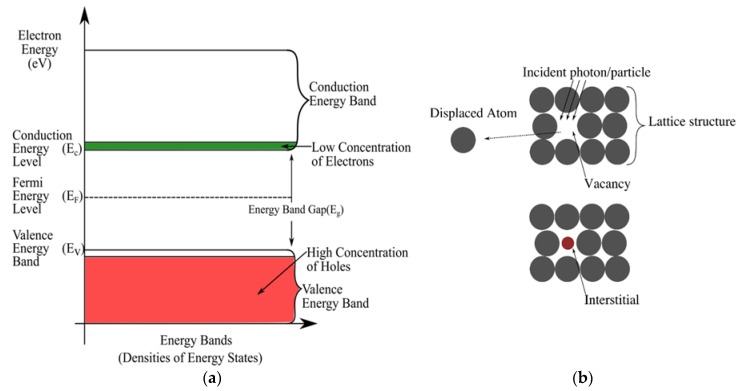
(**a**) Semiconductor Energy Bands; (**b**) lattice structure Vacancies and Interstitials.

Silicon being a group four element, comprises of four valence electrons in its outer most shell. This way, a silicon atom makes covalent bonds by sharing electrons with the neighbouring atoms. The sharing could be among the silicon atoms themselves or even atoms of a different element - through the doping process [13]. Therefore, pure/intrinsic silicon is almost incapable of conducting current naturally [13,27]. It has gotten few/no free electrons in the conduction band but has gotten more holes in the valence band as shown by Figure 2a [13]. Nonetheless, conduction could still occur due to crystal defects or thermal excitation [27]. Current electronic applications mostly deploy doped silicon. In doped silicon, the sharing atoms are from either group 3 or group 5. During the bonding process, electron sharing creates an electron deficiency; if the sharing atoms are from group 3, or an excess of electrons; if the sharing atoms are from group 5 [13,27]. A deficiency creates holes [13] which are free and move around just like the excess electrons. After the bonding process, the loss of excess electrons leaves a positive charge on the impurity atoms while the gain of an electron leaves a negative charge on the impurity atoms. Therefore, a region where there is an excess of the negatively charged electrons becomes an N region: N-type doped semiconductor, while a region with an excess of positively charged holes becomes the P region: P-type doped semiconductor [13,27].

**Figure 3:**
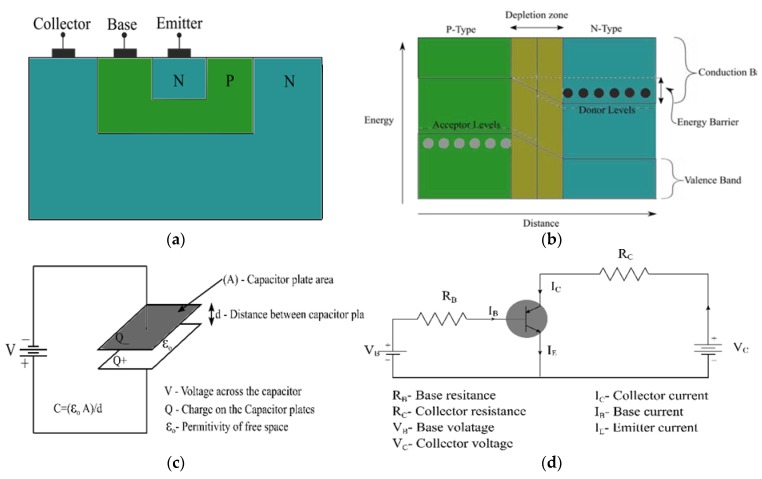
(**a**) Cross-section of an NPN transistor (similar to that of a photodiode); (**b**) an unbiased PN Junction; (**c)** a parallel plate capacitor; (**d**) an NPN transistor circuit diagram.

When the P and N types are in contact, electrons in the N region move across to the P region to make recombinations with the holes [13]. At this junction, the impurity atoms get depleted of charges that results in an electric field due to their positive and negative polarization. The electric field has an associated electric potential that ceases further charge flow across the junction. Therefore, this results in a necessity for an external potential, bias, for charges to travel across the junction [28]. This applied potential should be in opposition to the intrinsic region’s electric field potential. Charges are not only stimulated electrically with the potential bias [13] but also thermally [10,29], and optically [30]. PN junctions are the main components of semiconductor devices as shown in Figure 3a. 

### 2.2. Functionality and Operational Principle

The operation of LEDs is based on electrical stimulation/ electroluminescence; electrons gain electrical energy and drift towards holes that results into recombinations [13]. During these recombinations, energy equivalent to the semiconductor material’s energy band gap is dissipated off in the form of light photons [13]. There is also a direct proportionality between the electrical energy applied and the photonic light produced by LEDs, i.e., more current leads to more charge recombinations, hence, the production of more light and vice versa [13]. Transposition of these processes explains the working principle of photodiodes. Here, the charges receive energy in the form of electromagnetic radiation that makes electrons drift towards the holes. 

This charge flow creates an internal electric field that ceases the further flow of charge, hence, establishing an equilibrium in the depletion region of the PN junction. Therefore, an external positive or negative potential has to be applied against the newly-present internal potential; so that electrons and holes move across the junction again. This positive or negative potential is also termed as forward or reverse biasing depending on its orientation. In the forward biasing of a PN junction, the positive bias voltage repels the holes on the P side. Similarly, the negative bias voltage repels the electrons on the N side. These repulsions from both the negative and positive bias voltages make the charges overcome the internal electric field potential in the depletion zone (Figure 3b). This makes charges eventually flow over the junction, hence, the operation of a PN junction. In contrast, during the reverse bias, the positive bias voltage attracts the electrons from the N side while the negative bias voltage also attracts the holes from the P side; that results in no charge flow across the junction. 

Despite the reverse bias, some random charges can still overcome the electric field potential in the depletion zone. This random charge flow over the junction leads to a current flow termed as a leakage/dark current. This dark current flow during the reverse bias gives a clue to the intuition of radiation detection by a PN junction, i.e., medical radiation detection using a reversely biased PN junction. When photons fall on a reversely biased PN junction, their energy is absorbed by the electrons in the N region. Due to this energy absorption, electrons overcome the depletion region’s electric field potential thus formulation of recombinations. From Equation (1) [16], an electrical current *(I)* is a result of summing up (integrating) all the small charges, *(dq),* flowing per unit time *(dt).* The current *(I)* can also be defined as the rate of charge flow. An induced measurable photocurrent [31] can be used to depict the nature of photonic energy that induced it. The generated electrical signal may be of small magnitude or incompatible to the measuring device (ammeter/ multimeter). Therefore, a photocurrent may not be directly sensed by a detector device. In this case, amplification has to be done to produce a measurable electrical current [25,32,33]. Medical radiation detection using PN junction-based dosimeters exploits this principle to measure and characterize the quality and quantity of radiation striking the dosimeter; the characterization is based on the induced photocurrent.

(1) $$I=\frac{dq}{dt}$$

### 2.3. Present Literature

The ability of PIN photodiodes to detect mammography and radiology clinical beams are examined in a study by Romei et al. [9]. Here, reproducible linearity, system sensitivity, diode batch-to-batch reproducibility, and the correlation between diode read-out and absorbed dose are investigated using an S2506-02 photodiode [9]. A Monte Carlo simulation was also performed to investigate the effect of the photodiode casing during the measurement of low energy radiations. As highlighted in Section 2.2, there was a need for an amplification stage in order to acquire the signal in the current mode [9].

PIN diodes were also evaluated for detection of diagnostic radiology clinical beams while using a standard reference as calibration [34]. A linear correlation between the PIN photodiode read-out and the dose measured with standard dosimeters was observed [34]. In this study, satisfactory sensitivity; in accordance with the read-out dose values, small size, and being cost effective are highlighted as the advantages of using PIN photodiodes [34]. 

Further, a BPW34FS photodiode was benchmarked for; angular dependence-variation of the output signal according to the radiation beam angle of incidence, energy dependence, dose linearity, and sensitivity degradation (due to accumulated dose) using computed tomography X-rays [35]. Its response was then compared to those of the OP520 and OP521 phototransistors [35]. Air kerma/energy dependence response for all of the three devices implied the need for calibration for each device. All the three device signals were also highly dependent on the angle of incidence of the radiation beam [35]. The photodiode had low sensitivity that was unaffected by increased X-ray exposure [35]. The transistors’ high sensitivity also considerably plummets with an increased absorbed dose; this presented a need for calibration in order to obtain accurate results following continued exposures [35].

SFH206, BPW34, SFH205, and BPX90F PIN photodiodes were also examined for application as radio-protection detectors in radiology [14]. The examination was based on the minimum sensitive area (5 mm^2^), half angle (60°), and low-cost as comparison criteria [14]. This was because these devices are inexpensive and have a small volume [14]. Photodiodes are also defined as direct-reading real-time dosimeters, and their responsiveness to x and gamma rays, air kerma/absorbed dose linearity, and repeatability were analysed [14]. 

In addition, current mode S1223, BPW34, and PS100-6-CER2 PIN photodiodes were also appraised as real-time gamma radiation detectors while using photodiodes of different active areas and varying the number of diodes connected in parallel [15]. The assessment was done by deducing the photocurrent-dose rate, and accumulated charge-absorbed dose relationship [15]. A customized computer-based electrometer, that could measure photoinduced currents as low as 50 pA with considerably low errors, was also used. In this study, the photodiode’s induced current was linear with the dose rate while the accumulated charge was also linear with the absorbed dose [15]. The current response of the devices could be estimated with a function that is based on the dose rate and the photodiode’s detection volume, i.e., the product of the active area and depletion layer width [15]. 

### 2.4. Benefits, Limitations and Challenges as Potential dosimeters

Accurate detection and measurement of radiation-induced photocurrent is paramount and the ability to perform this is termed as sensitivity. Photodiode sensitivity can be enhanced by increasing the device’s sensitive area; to ensure more accurate results in medical radiation applications. However, this is related to an adverse effect of increasing the capacitance [14]. From Equation (2) [16], while assuming a parallel plate capacitor [18] with constant charge, the capacitance is inversely proportional to the applied voltage. It can also be observed from Equation (3) [15,16] that area-A (sensitive area) is directly proportional to the capacitance thus an increase in the area increases the capacitance. Therefore, due to the inverse proportionality between the capacitance and the voltage, there is an output signal-voltage amplitude drop associated with an increase in the area [14]. In other words, the parameter that affects the PIN photodiode’s photocurrent most is the active detection volume [28]. The detection volume is a product of the active area and the depletion region’s width [15]. While the active area is constant, the depletion layer can be varied by the amount of the reverse bias voltage [15]. This however increases the leakage/dark current that eventually affects the minimum dose rate (current) that can be measured [15]. A study by authors [14] also suggests combining the photodiodes in parallel (tiling) to increase the detection area. However, they further explicate that increasing the sensitivity this way has a cost of increasing the capacitance; hence a low voltage output signal.

(2) Q=CV

*Q*-Charge on capacitor plates, *C*- Capacitance of the capacitor, *V*-Voltage across the capacitor plates

(3) $$C=\frac{\varepsilon A}{d}$$

*C*-Capacitance, *ε*-Dielectric constant, *d*-Distance between the capacitor plates, *A*-Area of the capacitor plates

PIN photodiodes have a higher quantum efficiency over other current read-out sensors [15]. This infers that a higher fraction of the incident photon beam contributes to the photocurrent [14,36]. The PIN photodiode’s wide intrinsic region has high charge densities [37] that implies many charge carriers per photon that strikes this region [14]. There are no electrons filling the outermost energy bands in this intrinsic region, hence charges prefer filling up these empty energy bands; a phenomenon that results in a high charge concentration in this region.

Compared to ionization chambers, semiconductor detectors also have a higher signal-to-noise ratio, i.e., they require lower average energy to create a pair of charge carriers [9]. This could also imply high sensitivity to low energy radiation thus increase in the measurable range. Although photodiodes may give a small signal to radiation exposure, they are preferred because accuracy is crucial in dosimetry; photodiodes produce a pure signal with less noise [35]. 

Some attributes of an efficient medical radiation dosimeter include; having a wide dose-measurement range, high accuracy levels, giving a real-time response to radiation exposure, and being user-friendly [9]. The radiation-induced current of photodiodes only flows during radiation exposure, thus giving a direct measurement/output electric signal [14]. This implies that photodiodes are active and real-time dosimeters [15]. Therefore, photodiodes are preferred to passive dosimeters like the TLDs [15]. This is because the absorbed dose of the TLDs could be affected by fading during the time between radiation exposure and measurement; that leads to inaccuracy in measurements. 

Photodiodes can be termed as hard dosimeters because their loss of sensitivity after radiation exposure is negligible [35]. In other words, they have higher repeatability [15]. In dosimetry, repeatability refers to the extent to which the dosimeter gives stable/constant results following successive/consecutive radiation exposures - keeping all parameters constant [16]. This is a key feature for dosimeters since they are repeatedly used for dose measurements in different sessions. Therefore, the ability to give unwavering readings is pivotal. 

Since dosimeters measure the radiation received by the body, their structural composition relative to the human body should be taken into consideration. Silicon’s atomic number-14 is different from that of human tissue-approximately 7.4 [9]; therefore, both silicon and human tissue are associated with different chemical interactions. However, silicon may still be used for dosimetry of x and gamma rays, interacting with human tissue, by applying calibration factors [9].

PINs are vulnerable to atomic displacements in their lattice structure [38]. These displacements are due to absorbed dose/ photon energy of the incident beam as shown in Figure 2b. Increase in displacements could imply an increase in dark currents [15], but these damages in the PIN’s structure may be negligible. For instance, the lowest measurable photocurrent level for each sample in [15]’s study was more than two orders of magnitude higher than the nominal dark current before radiation exposure [15]. Considerably high dark current levels could, however, limit the minimum measurable level of radiation-induced photocurrent [15]. This is because the low energy radiations won’t be able to stimulate electrons and holes from the dosimetric traps [30]. Eventually, the post-recombination current signal will be weaker/lower than the pre-existing dark current signal: Unmeasurable.

Since an increase in the dark currents leads to a decrease in the photocurrent, periodic recalibration has to be carried out to ensure long term stability and accuracy. This is supported by the finding that states that the level of sensitivity loss as a function of radiation damage depends on the photodiode’s characteristics, the energy of the source, and the total absorbed dose [15].

Increase in ambient temperature also leads to a relative increase in the leakage current; the charge drift is stimulated by the heat energy. Yukihara [30] also observes a significant charge concentration (signal) decrease at room temperature. This is because the heat energy could easily stimulate the charges out of shallow traps. Recombinations of these charges from shallow traps produce dark currents; shallow traps are situated close to energy levels at the edge of the conduction and valence bands [30]. 

## 3. Phototransistors

Since photonic devices emit, detect, and manipulate light, devices such as metal–oxide–semiconductor field-effect transistors (MOSFETs), bipolar joint transistor (BJT), vertical double-diffused MOSFETs (VDMOSFETs), and transistors are not considered as photonic devices. Although this paper mainly focuses on photonic devices, this section also includes MOSFETs, BJT, VDMOSFET, RADFETs and transistors because they have the same structure, operation principles, and are often compared to photonic devices during medical radiation detection.

### 3.1. Structure

Phototransistors consist of a PN junction in the NPN format (Figure 3a)-similar to photodiodes and transistors. The only difference between photodiodes and phototransistors is that the phototransistor base section (P) is sensitive to the photons of light that strike it [11]. Since transistors are fundamentally amplifiers, they multiply the base current with a gain factor giving rise to an amplified collector current. Therefore, if the base of the transistor is photosensitive, amplified photocurrent signals will be produced as collector currents. Phototransistors are therefore defined as optoelectronic devices commonly constituting two relatively thick N-type sandwiching a thin P-type semiconductor material layer [39]. The NPN junction sections are emitter, base, and collector, respectively [16]. These sections are named according to the roles they play in electron transmission across the junction. In reference to Figure 3a, both the base and the collector are bigger than the emitter. The base inputs charge into the junction and on the other hand, the collector gathers charge out of the junction. Further, the collector is bigger than the base thus collects and takes into account all of the emitted charge without omitting any; this implies high sensitivity. Andjelković and Ristić also state that the base-collector junction is the sensitive volume of the phototransistor and is deliberately made longer than the base-emitter to achieve high sensitivity to the incident radiation [16].

### 3.2. Functionality and Operational Principle

Exposure of phototransistors to radiation stimulates the flow of a charge/photocurrent in them. This charge/photocurrent is a record of the type of radiation that induced it. Therefore, accurate quantification, evaluation and measurement of all collected charge guarantees accurate backtracking to the radiation that induces this charge/photocurrent. The product of the photocurrent and the time taken to collect this photocurrent is equivalent to the charge collected by the sensor during this time. This accumulated charge is proportional to the absorbed dose [15]. In addition, if there is a linear relationship between the intensity of the radiation-induced current and the dose rate, the intensity of the radiation-induced current is equivalent to the dose rate [15]. The radiation-induced photocurrent will be stable and proportional to the dose rate as long as the incident radiation dose rate is high enough during exposure [16].

### 3.3. Present Literature

Gamma radiation from a Co-60 source was detected with a collector-emitter biased NPN phototransistor [16]. The accumulated charge-absorbed dose relationship, dose rate effect on the induced current, short term repeatability, and stability of induced current were assessed. The radiation-induced current was stable with a less than 5% uncertainty and the induced charge was considerably good with a less than 3% uncertainty [16]. A notable current fall after an absorbed dose of 20 Gy was however observed; this was attributed to the radiation-induced lattice structural damages [16,38]. A non-linear relationship between the absorbed dose and the accumulated charge was also noticed; this was similarly due to the current gain damages [16]. The results of this study recommended the use of an NPN phototransistor because of its high sensitivity, linear induced current-dose rate relationship, and minimal dose rate dependence on charge sensitivity [16].

Radiation Field Effect Transistors (RADFETs) are p-channel metal oxide semiconductor transistors purposely manufactured for radiation detection. RADFETs were compared to a p-channel VDMOSFETs for radiation detection by analysing the radiation-induced threshold voltage alteration; the threshold voltages change after radiation exposure [40]. Here, the threshold voltage shift and radiation dose exhibited a linear dependence on each other when a +10v gate bias was applied [40]. The p-channel power VDMOSFETs responded more to radiation exposures than the RADFETs – there was a higher threshold voltage shift. This was attributed to more fixed traps that are induced by gamma radiation [40]. Fading was also examined at room temperature and it was more eminent in VDMOSFETs than in RADFETs [40].

In breast cancer radiotherapy treatment, bipolar junction transistors were tested as radiation detectors despite the fact that they are affected by accumulated dose structural damages [39]. Similar to the threshold voltage shift technique used in VDMOSFETs, this study uses the current amplification factor (*β*) shift to determine the absorbed dose – radiation absorption alters the value of *β*. Therefore, radiation is quantified and characterized according to the change in the value of *β*. In particular, a Darlington type BJT was used since it has a higher gain compared to other BJTs; its gain is a product of two BJTs-$\beta_{1}\beta_{2}$ [41]. This high gain ultimately implies a high response/sensitivity to low energy radiations [39].

Another study also tested the accuracy of MOSFETs in the detection of radiotherapy absorbed dose [42]. The MOSFET dose calculations were ascertained by referring to the Monaco and MasterPlan pre-treatment plans of different anatomical regions; this was aimed at ensuring synchrony between the planned and actual absorbed dose [42]. 

**Figure 4:**
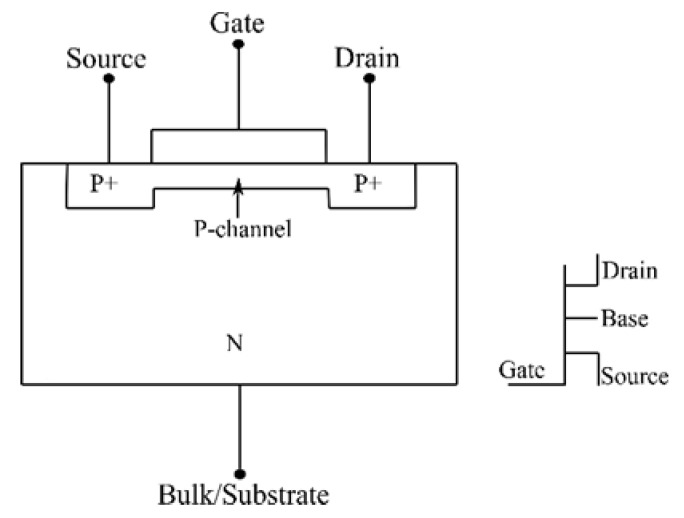
Depletion mode p-channel MOSFET.

RADFETs can’t provide real-time/ online dose information using conventional methods. Therefore, they were configured as a PN junction, i.e., the gate, drain and source terminals (Figure 4) were inactivated /grounded. Co-60 gamma radiation-induced current was thereafter measured from the bulk terminal [28]. The radiation induced-currents were stable and progressively increased with the increase in the bias voltage of up to 30V [41]. While the current increased with the dose rate in accordance with the power law, its read-out sensitivity was linear to the applied bias voltage [38,41]. This led to an overall performance that is relatively similar to that of the PN photodiodes; additionally, the device is unsusceptible to radiation-induced lattice structural damages [38,41]. 

While examining reproducibility, dose linearity, energy dependence, and fading, MOSFETs were investigated for in-vivo applications using an Alderson phantom [43]. The radiation-induced current signal was linear with applied doses between 0.2 and 2 Gy of the absorbed dose [43]. From the attained results, there was minimal temperature dependence between 22 and 40 °C, and the beam angle variation was within 5% [43]. The MOSFET’s in-vivo dosimetry was applicable in the 80 kV–250 kV range, and reproducibility was observed as a function of the absorbed dose [43]. The measured signal also faded/reduced as the time between radiation exposure and measurement increased [43]. 

### 3.4. Benefits, Limitations and Challenges as Potential Dosimeters

Santos et al. [44] demonstrated that phototransistors have good read-out stability for low energy radiation (Diagnostic range) [44]. However, they exhibit a high sensitivity loss during high energy radiation exposure; doses up to 100 Gy [16]. Furthermore, phototransistors have a higher current gain/higher sensitivity in comparison to PIN photodiodes, but they experience faster sensitivity loss with respect to the absorbed dose; this is due to the gain degradation [44]. In Figure 3d, the small base current $I_{B}$ is amplified by the gain factor of the phototransistor-*β*; *β* is the ratio of the collector current $I_{C}$ to the base current $I_{B}$. Typical current gains for phototransistors range from a hundred to several thousands [16]. A higher transistor gain factor, therefore, corresponds to the multiplication of the base current with a bigger value in order to obtain the collector current. This way, the phototransistor amplifies the small radiation-induced photocurrent, $I_{B}$, to a greater value, $I_{C}$, hence, higher sensitivity and good read-out stability for low energy radiation [44]. The main advantage of phototransistors over the photodiodes is this inherent current gain which brings the benefit of higher sensitivity to incident radiation [16]. 

This sensitivity can even be further augmented by increasing the bias voltage [45], i.e., the collector-emitter voltage. This consequently enhances the collection efficiency [16]. For investigating the emitter-collector capacitance, a parallel plate capacitor will be analogized. Since the emitter-base junction is considerably smaller than the collector-base junction, the emitter-collector capacitance will be assumed to be the collector-base junction capacitance [16]. The total charge on the parallel plate capacitor plates in Figure 3c is given by Equation (2) [16].

In Equation (3) [15,16], *ε* is the dielectric constant of the material between the capacitor plates. *ε* is equivalent to *ε_o_* if the dielectric material between the capacitor plates is air. Substitution of Equation (3) [15,16] in Equation (2) [16] makes the distance *d* between the capacitor plates directly proportional to the applied voltage *V*; with (*ε*A/Q) as the proportionality constant. An increase in the voltage across the capacitor, therefore, implies that *d* increases relatively. This thickness-*d* is related to the depletion layer of the capacitor - as stated by Anđelković [16]. In a study by Oliveira [14], this depletion layer implies the intrinsic region in PIN diodes [14]. Therefore, a wide intrinsic region implies many charge carriers per photon; this optimizes the device’s sensitivity. However, an increment in the depletion layer by increasing the voltage [45,46] is associated with an increase in dark currents; Figure 3 of Anđelković [16] clearly elaborates the dark current increment of various transistors [16]. Despite the fact that the transistors are of different brands, the dark current effects showed the same trend they appreciably increased with the increase in the collector-emitter voltage. Therefore, the increase in the bias voltage and structural damages increase dark currents. 

Similar to photodiodes, dark currents of phototransistors determine their minimum measurable dose rate [16]. For higher precision during dose rate and absorbed dose rate measurements, the difference between the measured current and dark currents should be as large as possible. This is because the induced current is the difference between the measured and dark currents [16]. Therefore, if the radiation-induced current is low, effectively differentiating it from the already existing dark currents will involve some inaccuracies. This implies that a phototransistor will measure only radiations that can produce photocurrents with values above the dark currents. Therefore, low energy radiations producing photocurrents below the dark current can’t be measured by the device; the induced currents are calculated by subtracting the dark current from the measured current [16]. Therefore, the difference between the measured and dark currents should be reasonably large to enhance precise current measurements [16]. A dark current can hence be termed as the “phototransistor’s induced-current threshold value” since only radiations that can produce currents above the threshold value can be measured. A phototransistor with a high threshold value has a limited measurable dose range, i.e., only radiations with enough energy to induce currents above the high threshold value will be detectable [37]. On the other hand, transistors with a low threshold value have a wide measurable dose range (high sensitivity). This is because low energy radiations can also induce currents above the relatively low threshold value, hence, being measurable. In practice, [16] states that the photovoltaic mode is preferred since it ensures a low dark current [16]. The low dark currents imply a low threshold value that increases detectable and measurable energy ranges, hence, high sensitivity.

In comparison to photodiodes, phototransistors are more susceptible to radiation damage. Therefore, phototransistors have a faster sensitivity degradation with respect to an absorbed dose; this is because of radiation-induced displacement defects in the Si bulk [16]. A displacement effect is when an incident radiation or particle dislodges a lattice atom from its normal location through the Rutherford/nuclear elastic and nuclear inelastic scattering processes. This results into a vacancy; non-existence of an atom in its lattice vicinity, and an interstitial; the existence of an atom in a lattice structure vicinity where it is not meant to be (Figure 2b) [36,47]. An increase in these defects implies an increase in the density of the recombination centres; this reduces the minority carrier lifetime [36,46]—time taken by a minority carrier to recombine. Consequently, there is a decrease of the current gain [16] because there was an increase in the leakage current; resulting from an increase in the density of the generation-recombination centres [48]. In other words, the creation of more recombination centres in the form of isolated and clustered defects [47] could be assumed to be diluting the charge concentration. This is because an increase in these centres will make the constant charges numbers to be less compared to the newly increased number of recombination centres. Therefore, charges will have more room to easily recombine from anywhere thus reduced minor carrier lifetime; this finally results into low currents. Despite this degradation due to the absorbed dose, phototransistors could still be used as dosimeters while applying a correction factor; because they have a higher sensitivity to radiation [35].

MOSFETs also consist of a PN junction, but they have a metal oxide layer as their sensitive/radiation detection region [28]. Therefore, MOSFETs with a thick metal oxide layer have more hole-trapping centres; this implies higher responsivity [49]. When both the MOSFET source and drain terminals are grounded, the radiation sensitive field effect transistor version of MOSFETs could be considered as two $P^{+}/N$ junctions connected in parallel [28]. MOSFETs are used in radiation detection, but the p-channel MOSFETs (RADFETs) are preferred over current mode dosimeters. This is because they are portable compared to diodes and photodiodes, and their sensitivity could be boosted with bias voltage applications – just like for diodes and photodiodes [28]. Similar to PN junctions [28], incident radiation on the metal oxide layer of the MOSFET immediately leads to the formation of electron-hole pairs [45]. This is followed by the attraction of all the electrons to the aluminium gate plate by the positive voltage bias; a condition that leads to a high hole concentration [40]. This ultimately makes the volume density of electrons in the inversion layer to be different from that in the substrate layer. The voltage obtained by application of the charge conservation principle between the gate and substrate semiconductor interface is termed as threshold voltage-$V_{th}$ [50]. This voltage can also be defined as the voltage at which the device operation commences [51]. After radiation exposure, a new equilibrium is established; this corresponds to a new threshold voltage value. A proper analysis of this threshold voltage shift could, therefore, be used to investigate the quality and quantity of radiation attenuated in the metal oxide hence this voltage shift. Applying the same principle, Sedra [52] uses the radiation-induced lattice damages as a parameter for dosimetry [39]; these damages reduce the gain factor of a transistor. A BJT Darlington transistor is used in particular due to its higher gain compared to other transistor types [52]. Here, the limitation of transistors-having high post-radiation exposure lattice structural damages is a benefit. This is because this limitation is used to depict the amount of radiation that led to the transistor gain factor drop. Therefore, higher gain drop implies more damages, hence, more absorbed dose and a low gain factor drop, on the other hand, implies less damage, hence, less absorbed dose. 

## 4. Photovoltaic sensors/Solar Cells

Photovoltaic sensors are devices whose operating principle is based on the photovoltaic effect. The photovoltaic effect is a process where incident photons of light excite valence electrons to higher energy levels/ conduction bands. The flow of these electrons in the conduction bands implies induction of a voltage and current in the device [36,53]. In medical radiation applications, it’s not only the light section of the electromagnetic radiation spectrum that excites these electrons to the conduction bands; the X-ray section can also stimulate electrons. 

### 4.1. Structure and Operational Principle

**Figure 5:**
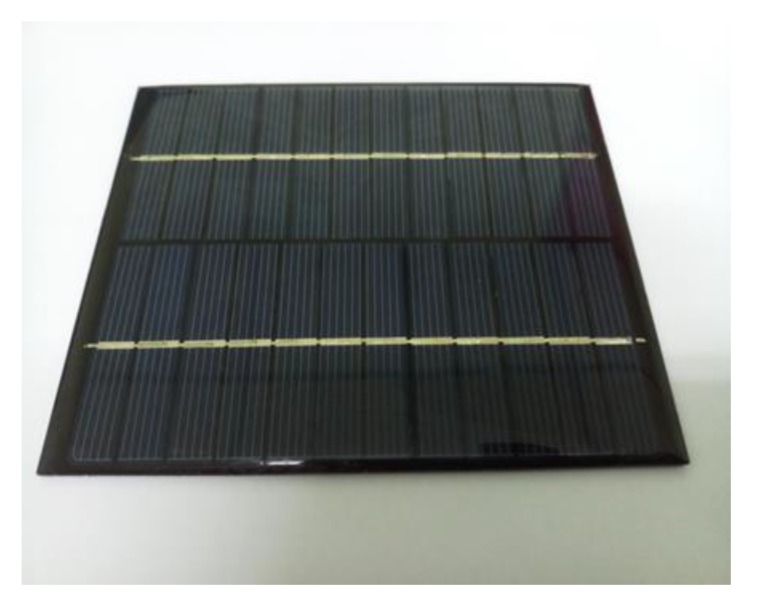
Solar Cell.

In reference to Section 2.1 of this paper, Gallium Nitride (GaN) and Cadmium Telluride (CdTe) are semiconductor materials. They are deployed in solar cells (Figure 5) to convert sunlight (electromagnetic radiation) to a current/charge which is stored as an electrical potential difference (voltage). Upon radiation exposure, semiconductor material electrons absorb the incident radiation energy and are excited to the conducting band (Figure 2a). Considering an unbiased PN junction of the semiconductor material in Figure 3b, these excited electrons could be localized in the N region. Therefore, their possession of radiation-gained energy enables them to drift towards the P section in order to recombine with the holes. In photovoltaic films, there is a non-equilibrium (free carrier concentration) created by illumination. This reduces the total volume of the depletion region while increasing the effective volume of the material where charges can be transported [2]. Photocurrents are also monitored with an application of a small bias voltage across the terminals of the device [2]. The drift of electrons implying a flow of a radiation-induced electric current, there is a conversion of radiation to a radiation-induced current. Therefore, a solar cell converts solar energy (radiation) to a storable form of electrical energy (charge).

A flow of $6.2\times 10^{18}\;$electrons in one second implies one unit of current-Ampere. This can also be defined as the flow of one Coulomb of charge per second. The quantity of induced current being directly proportional to the amount of radiation induce it, we can depict the type and quantity of the radiation that is inducing a photocurrent in solar cells – during medical applications today. Solar cells are grouped into three subgroups, i.e., first-generation wafer-based silicon; consisting of monocrystalline and polycrystalline solar cells, second-generation thin films; composed of amorphous silicon and CdTe thin film solar cells, and third-generation new emerging technology; including nanocrystal, polymer, and perovskite-based solar cells, dye-sensitized, and concentrated solar cells [54].

### 4.2. Present Literature

A GaN thin film was adopted in the detection of 40–300 kV Bremsstrahlung sourced X-rays while simulating the X-ray imaging of the index finger and wrist of the human phantom [2]. The film showed a high current gain, minimal radiation angular dependence, high sensitivity to X-ray intensity and had a linear total dose response [2]. It could also measure the 1 μGy $s^{-1}$–10 mGy $s^{-1}$ air kerma range with a signal stability of 1% [2]. Additionally, there was no need for geometry/ energy recalibration because the results varied with only a percentage of 2 – within the measured range of energy in the study [2]. Further, in this study, the solar cell executed high-resolution X-ray imaging since solar cells normally have a detection volume smaller than $10^{-6\;}cm^{3}\;$[2]. With some more enhancements, they would also be used in vivo biosensing [2].

A semiconductor monocrystalline silicon solar cell’s response to cobalt-60 gamma radiation dose was also studied using the thermal luminescence glow peak technique [10]. To navigate the sources of errors in gamma radiation dose measurements, both the solar cell and TLDs were used for radiation detection. According to the results, gamma dose measurements were more accurate while using solar cells [10].

Because of their low operational power and low cost, thin film sensors connected to data acquisition electronics and wireless data transmissions were used in kV and MV photon beam detections [33]. Their sensitivity per unit area was compared to that of normal photodiodes, and to that of an Electronic Portal Image Device (EPID) [33]. The thin films were placed under block sheets of a solid water slab phantom, and radiation exposures were made while varying parameters such as beam energies, dose rates, total doses, depths, and radiation exposure angles [33]. Further, IMRT sensitivity and precision tests like closed Multi-Leaf Collimator (MLC) were performed [33]. The detector’s performance was not dependent on the amount of the absorbed dose and the dose rate. The sensor’s sensitivity was also sufficient, i.e., a stable and accurate read-out signal was detected during the radiation exposures; hence, being a possible quality assurance tool [33]. 

Photovoltaic CdTe semiconducting thin films were also used for detecting diagnostic radiology and radiotherapy keV and MeV energies – imaging dosimetry energies associated with image-guided radiotherapy techniques (IGRT) [8]. The films provided real-time tracking of the tumours in IGRT treatment delivery [8]. The films further facilitated noise reduction and better image resolution compared to the commercially available indirect electronic portal imaging devices (EPIDs); these EPIDs are mainly made of amorphous/non-structured silicon [8,54]. 

### 4.3. Benefits, Limitations and Challenges as Potential Dosimeters

GaN thin films are almost independent of the angle of radiation exposure. This implies that the films will be able to give accurate dose measurements independent of the beam direction; thus, a wide range of geometric applicability [2]. Therefore, the application of GaN films in radiotherapeutic beam fields is feasible; the angles of these fields are selected while ensuring maximum dose delivery to the targets, and at the same time sparing the organs at risk (OAR) [4,55]. Therefore, the ability to efficiently measure the absorbed dose at all angles doesn’t present a need to alter the radiation field angles in order to suit the measurable dosimetry angle of the thin film dosimeter. In case beam alteration is done to suit the film’s dosimetric range, there may be increased radiation exposure to the OARs. These films could, hence, stage a better performance when compared to ionization chambers that are associated with a large angular dependence [56]. However, considering the geometrical shape of these films, they aren’t symmetrical like the ionization chambers. Therefore, there may be a variation in the results displayed from the different angles and positions of radiation exposure [33].

Even after frequent consecutive radiation exposures, the GaN thin film-based dosimeter’s radiation-induced current varied within only a 2-percentage range – hence, consistent results. Therefore, there is no need for GaN film recalibration; a procedure that is normally applicable to dosimeters in medical radiation [2]. This consistency may be due to fewer vacancies and interstitials [10,47,57] created in the GaN lattice structure – hence, insusceptibility to radiation-induced damages [58]. In other words, the films ‘accumulated-dose related errors are minimum, i.e., they produce consistent results. Due to the radiation hardness of GaN, its usage in tracking detectors affected by luminosity has also been suggested in space science and astronomical applications [59]. This radiation hardness is eminent in Figure 3a of Hofstetter [2]. In Hofstetter’s study [2], the photocurrents before and after radiation exposure are equivalent when plotted on the same graph. This implies that there were trivial photocurrent degradations due to radiation exposures [2].

GaN thin films with detection volumes of smaller than $10^{-6}cm^{3}$ imply a high spatial precision [2]. These films could be further developed for not only X-ray detection but also other radiotherapy treatment modalities such as the Stereotactic Body Radiation Therapy/Stereotactic Radiosurgery (SBRT/SRS) and Intensity Modulated Radiation Therapy (IMRT). These treatment modalities involve the delivery of high doses to relatively small dynamic fields in a short period of time [4]. SBRT and SRS volumes are normally small and therefore a correspondingly small volume dosimeter would be vital in the replication and simulation of these treatment fields during dosimetry. Since these films are relatively thinner and more flexible than present dosimetric films, they could also be easily applied in dosimetry applications that involve curved surfaces where normal dosimeters cannot easily be applied [33].

The band gap energy is proportional to the amount of energy of a photon released after recombination of an electron and a hole [13]. CdTe’s bandgap being in the 1.44–1.47eV range [8,54], and that of GaN being 3.4 eV [60], CdTe and GaN-based films can be operated at room temperature [60,61]. Therefore, CdTe-based dosimeters will have less temperature dependence because the room temperature’s heat energy may not excite electrons from the valence band to the conduction band; the excitation consequently results into a photocurrent. Therefore, a fairly huge proportion of the induced current would be as a result of the electromagnetic radiation incident on the detector. This implies higher accuracy and quantum efficiency levels of the dosimeter. Nevertheless, a large band gap also insinuates that only radiation whose energy is above this band gap will be measured. This is because the band gap energy is required to pluck an electron from the valence band to the conduction band; so that an electron drift occurs in the conduction band, i.e., a photocurrent (Figure 2a). Therefore, low energy radiation whose energy is below the band gap will have less probability of being accurately detected. This may limit the dosimeters from measuring low energy radiations that affects their sensitivities. Contrarily, some studies have reported the detection of some photocurrents induced by radiations whose energy is below the band gap [60]. For incident radiations whose energies are above the band gap,$\;10^{3}-10^{4}\;$range gains were observed [60]. In addition, since shallow and deep traps are situated between the valence and conduction energy bands, a wider band gap may also imply higher sensitivity because charges will get easily stuck in these multitudinous traps. The magnitude of the radiation energy used to free these charges is proportional to the number of freed charges; this is proportional to the induced photocurrent [10]. Presence of these numerous traps could also lead to fading where the charges can easily be stimulated out of these traps. The stimulations could be by, for example, heat energy at room temperature. There will thus be inaccuracies in the signal detected. This is because all the trapped charges are a “record” of the energy used to trap them [10]. Due to the numerous traps containing charge, less energy would be required to stimulate charge out of any of these traps. Ultimately, there will be inaccurate backtracking of the amount of energy that was involved in the trapping of these charges.

GaN thin films are associated with large charge gains where free charge carriers are generated due to incident radiation. This increases the number of free charge carriers available to transport charge; a phenomenon known as photoconductivity [60]. Photoconductivity leads to more charge flow and a high non-linear photocurrent flow [60]. This results into an amplified radiation-induced current produced by the detector; a slight change in the kerma can readily be observed under typical operational conditions [2]. This photoconductive model could also be referred to as conductivity modulation (carrier density) because of the photogenerated carriers [60]. This model produces more photoconductive gains that are in the order of $10^{3}-10^{4}$ for GaN sensors [60]. These gains are high compared to the detection process where there is direct charge extraction [2]. In addition, there is a linear relationship between these current gains and the amount of radiation incident on the dosimeter-thus being linear for the 0.5–2 Gy dose range [10]. Despite blockage of all the ambient light and operation of the detector at room temperature, photoconductivity produces dark currents, i.e., a signal will be detected even before any radiation strikes the dosimeter [2]. 

CdTe films are cost-effective [54] and are associated with a direct detection method–thus low noise effects [8]. GaN films also have a high sensitivity to noise ratio and they are nearly independent of the air kerma rate [2]. A GaN film detector produced direct signals that were not processed using any configuration formula/measurements; hence, faster and simpler read-out compared to traditional detectors [2]. In addition, the induced photocurrent is stable with a standard deviation of ±0.028 μA; the current varied within a range of 1% during 10 min of radiation exposure [2]. This is as a result of very slow non-exponential photoconductance decays; these decays are associated with carriers being trapped in shallow and deep traps [30,60]. The stability of this current could also imply reproducibility; repeated series of experiments produce the same results while maintaining a constant set-up. GaN sensors were observed to be reproducible, i.e., there was an average standard deviation of 1.3% in five repeated series of measurements carried out by Hofstetter [2]. In another study [33], the variation of results of the individual photocells in a solar cell array-based prototype was within a percentage of one [33]. However, the reproducibility of the solar cells cannot be compared to that of diodes used in dosimetry; this could be attributed to errors during manufacturing [33]. 

## 5. Charge Couple Devices (CCD)/Charge Metal Oxide Semiconductors (CMOS)

When a capacitor is fully charged, charge flow ceases and there is a static charge stored on the capacitor plates and in the dielectric material. The capacitor is then referred to as fully charged because this trapped charge in the dielectric material, and on the capacitor plates, doesn’t flow unless a complete circuit is connected to it. For the capacitor in Figure 3c, the dielectric material is air, but any other material could be placed between these plates. This could boost the capacitor’s capacitance since the dielectric constant is dependent on the dielectric material. The dielectric constant is also directly proportional to the capacitance as seen in Equation (3) [15,16]. Capacitors, therefore, are energy-banks since they store charge equivalent to the voltage potential connected across them as observed in Equation (2) [16]. When the capacitor plates are metal, and the dielectric material is a metal oxide, a capacitor with a PN junction is formed. Therefore, electromagnetic radiation striking the surface of the capacitor plate induces an electron-hole pair; thus, conversion of the incident electromagnetic radiation energy to charge. A Charge Coupled Device (CCDs) is an array of single and independent metal oxide semiconductor capacitors closely packed in a sole block. The charge on each capacitor is transferable from one-unit cell/pixel /photo site of this block to another. Ultimately, the Analog to Digital Converter (ADC) transforms this analog current signal (charge) to digital format [62].

### 5.1. Structure and Operational Principle

**Figure 6:**
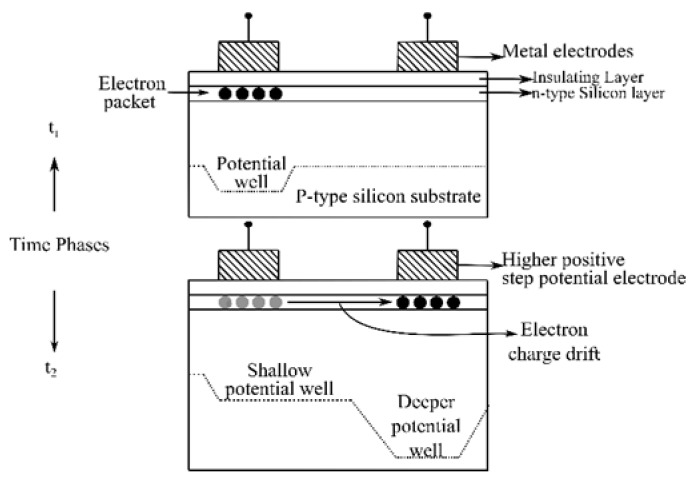
Electron Drift to deeper wells.

In a CCD, the actual charge transfer takes place in potential energy wells situated in *n* or *p* substrates that are found below electrodes; these electrodes are connected to a multiphase pulsed clock voltage [62] (Figure 6). Biasing one gate electrode with a positive step potential leaves the adjacent electrodes at a lower voltage [62]. This, therefore, creates a deep potential well below the biased electrode [62]. In this potential well, the electron charge is trapped and stored as seen in the first-time phase $t_{1}$ of Figure 6 [62]. However, if these adjacent electrodes are also consequently biased with a higher positive potential, there will be deeper potential wells below the adjacent gate electrode [62]. Since electrode charge carriers prefer lower energy for stability, there will be a drift of charge to the newly created deeper wells as illustrated in the second time phase $t_{2}$ of Figure 6 [62]. These wells could also be filled with electrons, which are not induced by radiation—heat induced electrons. Therefore, the charge signal can be stored for a short time; this time is much shorter than the thermal relaxation times for metal oxide capacitors [62]. This time normally ranges from 1 s to several minutes at room temperature; varies depending on the structure and fabrication process [62]. 

When the pixels are exposed to electromagnetic radiation, a charge; whose amount is a linear function of the illumination intensity, is accumulated from the electron-hole pair [62]. This charge is transferred and converted to a small voltage that is amplified in order to be compatible for analog to digital conversion by the ADC [62]. The amplified voltage is still susceptible to noise such as heat energy that also contributes to the electron-hole pair stimulations [62]. Complementary Metal Oxide Semiconductor Devices (CMOS) similarly operates under the same principle as the CDDs but their accumulated charge is amplified at each cell unit/pixel by multiple chip amplifiers, respectively [62]. Advantages of CMOS over CCD include; low power consumption, being inexpensive and easy to manufacture [63]. On the other hand, CCDs merits have; fast speeds, high dynamic ranges, greater light sensitivity and produce high-quality low noise images [63]. Factors such as camera size and noise [64,65] could also be a device preference and selection criteria. 

CCD pixel numbers range from 128–16 million [62] and it’s selected by the manufacturer and hence, they determine the level of sensitivity of the device.

### 5.2. Present Literature

Radioactive tracers such as Iondine-131 ${(}^{131}$I), Yttrium${(}^{90}$Y), and Fluorodeoxyglucose (FDG) –${(}^{18}$F), are radionuclides that decay by emitting charged particles like electrons($\beta^{-}$), positrons($\beta^{+}$) [57,66], and gamma rays at speeds surpassing the speed of light in that particular medium/tissue [66,67,68,69,70,71]. This phenomenon polarizes the medium, and photons are emitted after a molecule retains its normal/stable position [68,69]. In other words, the excited electrons fall back to their ground states due to the fading of the polarization effect [66,68,69]. The photons are emitted as an electromagnetic radiation wave front [69]–Cherenkov radiation (λmax ∼180 nm; water [68,72]). When Cherenkov radiation is accurately quantified and detected using CCD detectors [70], it depicts information regarding its source. For instance, the Cherenkov radiation could be emitted by anatomical structures, tissues or even cells [57,73]. The speed at which light travels through a medium is proportional to the refractive index of the traversed medium; Cherenkov Luminescence (CL) emissions increase as the refractive index increases [69]. Therefore, different media will produce different CL emissions. The solid state of matter has particles closely packed together and therefore if radiation or a particle is travelling through it, there will be more interactions between the photons of the radiation/particle and the solid particles. Therefore, there are more interactions in solids than in liquids and gases; the speed of light in fluids is higher than that and in solids. However minimal these interactions may seem, they reduce the velocity of the light; the reduction in the speed of light has a positive correlation with the refractive index, *η*, of the medium [69]. With the *η* of water being 1.33 [66] and that of biological tissue being ∼1.36–1.45 [66,74], the velocity of light, $v_{m}$, in these media is ∼0.75 c and ∼0.7 c, respectively (Equation (4) [69,71]) [69].

(4) vm=cη

$v_{m}—$Velocity of light in a medium, η—refractive index of the medium, c—Speed of light in a vacuum

When a body moves with a speed relatively close to that of light, its kinetic energy will not be be given by the classical mechanics formula $-E=1/2mv^{2}$. The kinetic energy will be given by Equation (5) [69,71].

(5) E=mc2[1(1−v2c2)−1]

E—Energy of a particle moving at a speed close to that of light, c—speed of light, m—mass of the particle, v—velocity of the particle

If this energy exceeds a certain threshold energy value [75], the Cherenkov radiation will be produced [69]. We can substitute the threshold kinetic energy (0.511 Mev) [66] into Equation (5) [69,71] in order to calculate the minimum velocity that has to be possessed by a particle to stimulate Cherenkov Emissions (CEs). CEs increase with an increase in the refractive index of a medium (Equation (7) [71,75]); the threshold energy to induce the Cherenkov radiation is inversely proportional to the refractive index. However, CEs don’t solely depend on the refractive index but rather many other factors such as the density and geometry of the medium, and the type of radioisotope [66,71]. During radiation-tissue particle interactions, there may be some Bremsstrahlung radiation. Bremsstrahlung is the radiation that is dissipated after deceleration of a particle cutting through the Coulomb field of an atomic nucleus [66]. Bremsstrahlung would, however, have no effect on the refractive index of the medium during particle interactions [69]. 

The Cherenkov radiation principle has its applications in both diagnostic radiology; Cherenkov Luminescence Imaging (CLI), and radiotherapy; thyroid and the liver radiotherapy [57,66,68,69,70,71]. Despite the use of the Cherenkov radiation in the measurement of beta-emitting radionuclides starting as early as 1968 [76], CLI is a new modality in the nuclear medicine arena – it was recently initiated in 2009 [69]. Therefore, literature pertaining to CL in medicine is not much compared to other photonic devices, but it is progressively emerging. 

In one study [68], the CLI of β emitters and imaging agents characterized with both radioactive and a fluorescence emission were examined. First, the medical imaging applications of β emitters and imaging agents were each investigated separately. Later on, both the β emitters and imaging agents were combined in a hybrid technology during image-guided surgery [68]. Application of β emitters and imaging agents separately is associated with both some advantages and disadvantages. However, in a hybrid technology combining a β emitter and an imaging agent, the shortcomings of one technique could be compensated by the other. Nonetheless, further research still has to be done to ensure the accuracy and sensitivity of the attained signal. 

Radiotracers emitting high-energy positrons via post-decays were also used to detect CLI. Radiotracer elements are radioactive and their quantity and number of particles they emit reduce with time –according to Equation (6) [69]. In this study, CLI in vivo application was validated by investigating the signal/Cherenkov light emitted by radiotracers injected in mice [69].

(6) t12=[In(2) τ ]

t12—Half-life (Time for half the quantity of the radioactive nuclei to decay), τ—Decay constant

The viability of Cherenkov Emission (CE)-based portal imagers in dynamic and stationary CyberKnife radiotherapy treatment fields was investigated [70]. Using a half-full water tank and a circular radiation beam with a diameter of 60 mm, CEs were stimulated in tissue equivalent materials [70]. CCDs placed behind the tissue equivalent materials were then used to detect CEs stimulated by both dynamic and stationary radiation [70]. A comparison with the results from an onboard linac portal imager was done to reveal potential resolution and contrast limits [70]. Results showed that the CE-based technique’s contrast percentages through both air and water were lower than those of the linac-based portal imaging system [70]. In all the above applications, Cherenkov Luminescence was emitted after a particle(s) travelled through a medium. From Equation (5) [69,71], the velocity of the particle must exceed the velocity of light in a given medium. Let’s now assume a particle whose velocity *v* is just equivalent to the speed of light $v_{m}$ in a particular medium. To attain its kinetic energy, we shall substitute Equation (4) [69,71] into Equation (5) [69,71] that will give rise to;

(7) E′=mc2[1(1−1n2)−1]

Equation (7) [71,75] therefore represents the amount of kinetic energy the particle has if it is traversing the medium at a speed of light in the medium. Since the particle velocity has to surpass that of light in the same medium, the particle with energy $E^{\prime }$ will not produce the Cherenkov radiation. This is because the light photons also have this same energy while travelling through the medium. Therefore, a Cherenkov particle has a velocity v greater than $v_{m}$ i.e., $v>v_{m}$. Thus, the Cherenkov particle’s energy $E_{Cherenkov}$ is greater than $E^{\prime }$($E_{Cherenkov}>E^{\prime }$). With this, we could now define $E^{\prime }$ as the minimum energy that a particle must be having above which it will emit Cherenkov radiation. Hence, $E^{\prime }=E_{Threshold}$ which is the minimum energy that a particle is supposed to attain before it emits a Cherenkov radiation. With the refractive index of water being 1.33, $E_{Threshold}$ is 0.264 MeV and if the refractive index of tissue is assumed to be 1.4, $E_{Threshold}$ is 0.219 MeV [71].

### 5.3. Benefits, Limitations and Challenges as Potential Dosimeters

Since CCDs are luminescence-based detectors, they can yield inaccurate results from the luminescence emitted by the used fluorophore [66,68,71]; the fluorophore brightness affects the signal intensity. The CE brightness is affected by several signal depletion factors such as excessive luminescence attenuation by tissues that have chromophore absorbers like hemoglobin; tissues are also heterogeneous in regard to their refractive indices [66,68,71]. Scattering effects, ambient light, electrical noise by camera dark currents, and surface reflections could also affect the signal [66,68,71]. All these factors consequently reduce the final signal magnitude and the image resolution. This signal could, however, be boosted by using liquid scintillators in conjunction with CLI imaging; as one hybrid technique [66].

CDDs or CMOSs detect the wavelength of CLs close to that of visible light (Ultra Violet (UV)). This implies that other light sources produce noise effects; CLI should be performed in total darkness to increase the detection efficiency of CCDs [66,68]. Ambient signal detection could also be eliminated by synchronizing the linac pulses with the CCD camera capturing time intervals [71]. 

The signal detected by the CCDs is directly proportional to the CL intensity. Further, the CL intensity is also directly proportional to the half-life (Equation (6) [69]) of the radioisotopes that stimulate the luminescence [68]. CLs measured by CCDs are, therefore, of low spatial resolutions compared to fluorescence luminescence. This is because CL is an indirect (secondary) luminescence; high-speed moving charged particles polarize molecules of a material–primary CL stimulation stage [68]. Since different radioisotopes emit decay particles with varying magnitudes of energy, there will also be a variation in the CEs generated; the radiation intensity reduces due to the half-life effects [66]. Further, with reference to Equation (7) [71,75] CE luminescence is refractive index dependent. Therefore, CEs will fluctuate according to the tissue refractive index variation [71]. This implies that there will be no homogeneity in the intensity of the signals attained. In turn, some regions in the image will appear brighter and clearer than the others due to more luminescence. Similarly, other regions will appear darker due to less luminescence in those regions. However, studies have been carried out and results have shown that CLI imaging could be used to increase the spatial resolution of SPECT images [71].

CCDs have high quantum efficiencies in the ∼570–720 nm spectral range [68]. This makes CCDs feasible in the imaging of common luminescent molecules [68]. However, CCD efficiency declines to half when the imaged wavelength is less than 350 nm; yet this is a region where CL is highly concentrated [68]. Despite the fact that the efficiency decline effect is less in near-infrared dyes, there is consequently deterioration of the CCD signal [68].

CCDs are also less likely to take real-time measurements since they involve the use of visible light [69]. Visible light has a low penetration power because its wavelength is more than 450 nm. This implies the need for proper exposure of the areas of interest before any imaging is done. This way, image acquisition could take approximately 5 min [68,69]. However, this operation time is still quite short compared to other imaging modalities like Positron Emission Tomography (PET) where it takes about 20 min to acquire the same images [69]. Longer treatment times/data acquisition times could, however, increase susceptibility to errors resulting from patient movements; hence, the need for additional patient immobilization procedures. This extra immobilization process is likely to cause fatigue in patients.

When CE-based images were compared to EPID images [70], CE demonstrated an almost similar contrast for imaging of an air-water inter-surface lining [70]. On the other hand, EPIDs had a higher image contrast for the air and water media inter-surface lining—during a real-time video [70]. Cherenkov luminescence-based video imaging during a breast cancer intra-surgery also increased precision in the patient positioning [71]. 

Novel techniques like CE-based imaging could also improvise for lack of EPIDs on devices like CyberKnife®. This would improve patient immobilization/positioning in radiation therapy; hence, accurate tracking of the targets (tumours)—conventionally achieved using portal imaging (EPIDs) [66,70]. CE-based imaging would be moderately cheaper compared to the normal imaging tools that are currently used in medical radiation imaging [66,71]. 

CL intensity depends on the speed of the particle; this speed is determined by the half-life (t12) of the radioisotope [69]. When the quantity of a radioisotope reduces to half its initial amount (after the half-life), the CCD signal intensity will similarly dwindle [68]. To ensure a low patient absorbed dose, radionuclides with short half-lives are commonly used. However, larger quantities of radioisotopes having short half-lives could be administered in order to attain a prolonged stable signal. This ultimately leads to excessive radiation attenuation by tissues [3,4] and high diagnostic patient doses [68]. Lack of sensitivity stability over a prolonged duration could limit the application of the CLI imaging technique to procedures like total body tomography; imaging of the whole body is done—not only a specific region of interest [69]. 

Although Cherenkov luminescence emits a very weak signal, if a highly sensitive camera is used, higher quality images may be obtained; this could be an alternative to the PET and SPECT scanners [57]. In addition, PET and SPECT scanners are relatively less efficient in the imaging of some radionuclides like yttrium-90 [66] that are used in radiotherapy [57]. Development of modalities such as radio-immunotherapy could also be facilitated; these modalities use radionuclides like yttrium-90 [57]. In addition, CLI could be used to validate SPECT images because it has a better spatial resolution compared to PET; during imaging/ display of positron-sourced proton distributions [66,71]. CLI is also currently a basis for the development of other novel techniques such as the Integrated Monte Carlo code; manipulates principles of both ionizing radiation and optical photons, Cherenkov Luminescence Tomography (CLT), and Cherenkov Luminescence Endoscopy [57,71].

Scatter radiation particles move at slow speeds, i.e., they have low kinetic energies. Therefore, they do not satisfy Equation (5) [69,71]; hence no CE illuminations will arise from stray particles [70]. This implies less need for noise filtration procedures [70]. In other words, there would be low noise signals when the radiation source is due to the Cherenkov luminescence since it doesn’t involve external optical stimulation [68]. 

With the promising CCD camera benchmark results as detectors for Cherenkov imaging in Table 1, we could further expound this phenomenon to estimate the absorbed dose. Cherenkov emissions were observed during radiotherapeutic radiation exposure of a human phantom with X-ray beams—a procedure that involved an absorbed dose of 5 Gy [77]. In this case, a specific Cherenkov image resolution would be correlated to a particular absorbed dose quantity involved in the production of the Cherenkov image (Table 2). Therefore, CCDs would be applied as indirect dosimeters where an image resolution would be calibrated to imply an absorbed dose value.

## 6. Summary

Although there may be a multitude of photonic devices applied in medical radiation dosimetry, this paper has focused on mainly photodiodes, phototransistors, photovoltaic sensors and CCD cameras. This is because they are; currently the most commonly applied in medical radiation detection based on the literature available, relatively low-cost devices, and there are no complex systems associated with their applications. CCDs have been in implementation for approximately a decade [69,71]. Therefore, CCDs could have more medical application deficiencies and less literature pertaining to their usage compared to other photonic devices. Such new technologies: Cherenkov imaging using CCD cameras, and the margining of fluorescence dyes and Cherenkov radiation exposure as a hybrid technology (in form of a single-imaging modality) present better results. However, further studies have to be carried out to increase CCD efficiency while minimizing their related hazardous effects. This would facilitate a quicker approval by governing and implementation bodies; hence, more animal and preclinical trials [57]. This will stimulate more clinical trials that will finally improve the results of these applications [57].

As a discrete analysis, photodiodes and phototransistors have relatively more frequent applications compared to the CCDs and solar cells; based on the present literature and studies. Photodiodes like the BPW34 have been associated with more pros as medical radiation detectors in this review-Table 1. They have a high quantum efficiency, low dark currents, and low radiation-induced structural damages. These characteristics could be considered to be some of the cornerstone characteristics of ideal dosimeters [15]. These features further suggest that these devices are durable when applied as radiation dosimeters. This is because there is less need for correctional/calibrations while using the BPW34 [35]. This implies that they cannot easily wear out due to repeated usage. Being resistant to radiation-induced damages is an essential feature in radiation dosimetry that may guarantee accurate and stable results even after repeated usage; the long-life span of a dosimeter could also imply durability [35].

On the other hand, phototransistors are susceptible to radiation-induced damages despite having a relatively high amplification capability [35]. Therefore, calibration and correction procedures will have to be carried out to maintain the device accuracy; this implies a short lifespan for these devices since there is degradation and wear out with increased radiation exposure [35]. Users will have to consequently buy new devices after numerous calibrations on old ones. On the other hand, photodiodes can be used for quite long durations with less or negligible wear out. Durability could thus also be considered a key characteristic of dosimeters associated with accurate results even preceding multiple applications. 

This presents photodiodes as fairly more robust compared to phototransistors, solar cells and CCDs. This is because they produce low dark currents, have fairly higher insusceptibility to radiation-induced structural damages and are negligible post-radiation sensitivity loss [15]. However, more studies have to be carried out to further resolve their flaws like low sensitivity to radiation and having a narrow measurable range. Alternatively, applying a solid-state scintillator in front of the photodiode could resolve its low sensitivity to the detected radiation [79]. This may perhaps make them to be more quintessential dosimeters in medical radiation. 

The displacement effects of these photonic devices take place in the lattice structure as illustrated in Figure 2b. Therefore, phototransistors could be fabricated using elements more resistant to radiation-induced displacements – such as those of solar cells or even better ones. More research on how to make the lattice structure of semiconductor devices more radiation hard will resolve this setback. Radiation-induced displacements occur in almost all photonic devices used for radiation detection; the magnitude of these damages could vary from one device to another. Photodiodes with inbuilt gains/amplification could also be fabricated. Alternatively, better amplification techniques could be applied to photodiodes in order to improve their signal magnitude and quality. In Hofstetter’s Figure 3b [2], solar cells are shown to be radiation-hard. Therefore, further exploitation of this characteristic may also make them ideal radiation detectors. During photonic-device based radiation detections, ambient light and temperature/heat buffers should be applied; an appreciably high spurious signal may be detected due to these factors.

In medicine, radiation is mainly used in diagnostic radiology and radiation oncology. Diagnostic procedures such as bone densitometry use significantly low radiation doses to produce X-ray images. On the other hand, high dose radiotherapeutic treatment modalities such as stereotactic radiosurgery (SRS) could impart absorbed doses of approximately 8–18 Gy [80,81] in one fraction. This implies that the photonic devices to be used for medical radiation detection should measure radiation doses from about 0 to 18 Gy in one exposure/fraction. Table 2, therefore, shows the dosimetric ranges of photonic devices in order to determine their feasibility for medical radiation dosimetry applications. 

From Table 2, photodiodes could measure minimum and maximum air kerma doses of approximately 0.001 cGy and 8.3 cGy, respectively. In addition, the table illustrates that photodiodes could be implemented in diagnostic radiology procedures like CT scans and X-ray imaging since their relative absorbed doses lie in the photodiode’s measurable range. Therefore, radiotherapy dosimetric procedures involving relatively higher absorbed doses may not be effectively implemented with photodiodes. Instead, phototransistors (MOSFETS) and photovoltaic sensors could be preferred since their reviewed dose range is 0.340–18,500 cGy; which is suitable for radiotherapeutic dose measurements. For a Cherenkov emission to take place, the threshold particle energy ($E_{Threshold}$) has to be surpassed. This threshold energy could perhaps be surpassed in applications involving higher energies and doses. In other words, CLI does not involve low absorbed dosages and energies. CCD cameras could, therefore, be calibrated and developed to measure absorbed doses in relation to a specific CLI image resolution. In this aspect, CCDs can be possibly designed to measure high radiotherapeutic doses. 

Solar cells are reasonably hard to absorbed dose-related wearing [2] in addition to their fairly broad dosimetric range of 0.086–500 cGy as observed in Table 2. Therefore, it would be more appropriate for dosimetry compared to the phototransistor that are greatly affected by radiation-induced damages. The phototransistors (MOSFETs) would have been the optimum choice due to their wide measurable dose of 0.340–18,500 cGy but are impaired by their appreciable displacement damage effects. 

Since cost also determines the application/preference of a specific device, Table 3 gives an insight into the probable prices of some of the photonic devices reviewed in this study. 

Fabricating phototransistors using elements more resistant to radiation-induced displacements would be the principal solution to their radiation-induced damages. This solution can, however, be implemented by only the device manufacturers. Since signal amplification using scintillators would perhaps resolve the photodiode’s low measurable dose range, photonic device users/researchers may resort to using photodiodes for diagnostic radiation detection. Similarly, researchers may enhance the solar cell’s measurable dose range using scintillators/signal amplifiers. Photodiodes and solar cells are therefore the most promising devices for detecting diagnostic and radiotherapeutic radiation, respectively. In nuclear medicine, CLI would be enhanced by mitigating light, heat and other ambient signals. Alternatively, higher resolution/sensitive cameras would also be used for CLI. However, as shown in Table 3, these cameras may be costly.

Photonic devices are generally promising radiation detectors, but more research has to be carried out to resolve or alleviate grave flaws like lattice structure displacements that mainly hinder their reproducibility and repeatability dosimetric parameters. Execution of more successful studies addressing the various drawbacks highlighted in this review presents a potential for providing more resilient, reliable and accurate photonic dosimeters.

In general, these research/development trends could be focused on mainly improving the photonic device sensitivity to radiation. This is because the photonic devices are purposely fabricated for light applications. While enhancing the sensitivity of these devices, their measurable dose ranges could also be expanded. This would, hence, make the photonic devices applicable for both low and high radiation dose measurements.

## Figures and Tables

**Table 1:** Comparison of the devices based on some dosimetry parameters and benchmarks.

Dosimetric Parameter	Dosimetric Device			
	Photodiodes	Phototransistors/ MOSFETs	Photovoltaic Sensors/Solar cells	CCD/CMOS (In relation to CLI)
Susceptibility to post-radiation lattice structural damages	Low [35]	High [16,35,38]	Negligible [2,10,59]	Not Applicable
Post-radiation dark currents/Noise	Low [35]	High [16]	Low [2,8]	Low -noise [68,70]
Post-radiation sensitivity loss	Negligible [15]	High [16,35,44]	Negligible [33,58,59]	Radioactive half-life leads to signal loss [68]
Quantum efficiency	High [14,15,78]	Adjustable [16]	High [33]	High [68]
Angular dependence	High [35]	High [35]	Almost independent [2,33]	Not applicable
Reproducibility/Repeatability	High [9,15]	Varies with absorbed dose [16]	Feasible [2,33]	Low [66,70]
Sensitivity to radiation	Low/varies with energy [35]	High [16,45]	High	Low [69]
Read-out type	Real-time [14,15]	Indirect/passive [28]	Direct [8,54]	Indirect [68,69]

**Table 2:** Medical Photonic Device Dosimetric Ranges.

Medical Procedure Analysed			Tested/Reviewed Dose Range (cGy)			
Dosimetry	Radiation Type	Dose Type	Photodiodes/LEDs	Phototransistors/ MOSFETs	Photovoltaic sensors/Solar cells	CCD/CMOS
Diagnostic Radiology	X-rays	Air Kerma	0.003–0.450 [9]	–	–	–
	X-rays	Air Kerma	0.001–0.043 [34]	–	–	–
	X & Gamma rays	Air Kerma	0.006–0.400 [14]	–	–	–
Computed Tomography	X-rays	Air Kerma	0.340–8.30 [35]	0.340–8.30 [35]	–	–
Not specified	Gamma rays	Air Kerma	47.2–330 [15]	–	–	–
Not specified	Gamma rays	Air Kerma	–	10,000–50,000 [40]	–	–
Breast Cancer Radiotherapy	X-rays	Alderson Rando Absorbed Dose	–	200 [39]	–	–
Radiotherapy	X-rays	Anthropomorphic phantom Absorbed Dose	–	4,000 –18,500 [42]	–	–
Imaging	X-rays	Human wrist and index finger phantom (one pixel)	–	–	0.086 [2]	–
Radiotherapy	Gamma rays	Air Kerma	–	–	50–200 [10]	–
General Medical Dosimetry	X-rays	Solid Water Phantom	–	–	0.1–500 [33]	–
Radiotherapy	X-rays	Human Body phantom	–	–	–	500 [77]

**Table 3:** Some Photonic Device Retail Price Ranges.

Photonic Device	Approximate Unit-Price (USD-$)	Online Store
**Photodiodes**		
S2506-02	0.0001–1.5	Alibaba
BPW34	1.6–2.45	Amazon
BPW34FS	1.98	Amazon
SFH206	1.6	Mouser Electronics
SFH205	0.79–1.92	Amazon
BPX90F	0.1–10	Alibaba
S1223	0.1–9.9	Alibaba
PS100-6-CER2PIN	78.38	Mouser Electronics
**Phototransistors**		
OP501	0.10–9.80	Alibaba
OP505A (Optek)	0.84–2.02	Amazon
BPW85 (Vishay)	0.10–18.80	Alibaba
OP521	0.001–10.00	Alibaba
Transistors		
BCV47 Darlington type BJT	0.20–0.23	Alibaba
MOSFETs	0.12–113	Mouser Electronics
**Photovoltaic Sensors**		
Solar cell	0.46–1.20	Alibaba
**Cameras**		
CCD	8.42–7000	Amazon
CMOS	9.99–6500	Amazon
